# Imported Tickborne Relapsing Fever, France

**DOI:** 10.3201/eid1111.050616

**Published:** 2005-11

**Authors:** Benjamin Wyplosz, Liliana Mihaila-Amrouche, Marie-Therese Baixench, Marie-Laure Bigel, Liliane Berardi-Grassias, Camille Fontaine, Michele Hornstein, Arezki Izri, Guy Baranton, Daniele Postic

**Affiliations:** * Hôtel-Dieu, Paris, France; † Hôpital de Mantes, Mantes-la-Jolie, France; ‡ Hôpital Avicenne, Bobigny, France; § Institut Pasteur, Paris, France

**Keywords:** Relapsing fever, Borrelia crocidurae, Borrelia hispanica, letter

**To the Editor:** Tickborne relapsing fevers caused by *Borrelia* species are characterized by >1 recurrent episodes of fever accompanied by headache, myalgia, arthralgia, abdominal pain, and eventually by hepatic or neurologic manifestations. In the Old World, *Borrelia duttonii* is endemic in sub-Saharan East Africa (*1*) and *B. crocidurae* and *B. hispanica* are distributed in West Africa and Mediterranean countries (*2*). In North America, *B. hermsii*, *B. turicatae*, and *B. parkeri* cause mild and sporadic fever cases, although several outbreaks have been reported (*3*). Relapsing fevers in disease-nonendemic countries are infrequently diagnosed and probably underdiagnosed (*4*). We report 3 patients with relapsing fever diagnosed in France in travelers from disease-endemic countries.

Patient 1, a 29-year-old French man, was admitted to Hôtel-Dieu in Paris for a fourth recurrence of a flulike syndrome. Three weeks earlier, he had traveled through Spain and Morocco, when high-grade fever, chills, myalgia, and arthralgia suddenly developed. Symptoms quickly resolved after treatment with salicylate and acetaminophen, but 3 relapses occurred within 20 days. He had a low-grade fever and persisting myalgia. Results of a clinical examination were normal. Analyses showed elevated levels of C-reactive protein (CRP) (368 mg/L), creatine kinase (269 IU/L), and lactate dehydrogenase (1,149 IU/L). Because of previous travel in Africa, Giemsa-stained blood smears were examined for malaria parasites. They showed helical bacteria suggestive of *Borrelia*. Polymerase chain reaction (PCR) of a blood sample and sequencing of the 16S rRNA gene identified this bacterium as *B. hispanica*. Cefotaxime was administered for 72 hours and replaced with doxycycline, 100 mg twice a day for 10 days. One month later, the patient was free of symptoms.

Patient 2, a 21-year-old Malian woman, was admitted to Hôpital de Mantes in Mantes-la-Jolie and delivered a normal baby with the gestational age of 36.2 weeks. One month earlier, she experienced a spontaneously resolving fever with myalgia. Biologic analyses of the mother showed anemia (hemoglobin 9.8 g/dL) with an inflammatory syndrome (CRP 164 mg/L). Giemsa-stained blood smears showed spirochetes. Molecular analyses identified these as *B. crocidurae*. Results of this analysis in the newborn were negative. The patient was treated with doxycycline, 100 mg twice a day for 10 days, and quickly recovered.

Patient 3, a 21-year-old Mauritanian woman, was admitted to Avicenne Hospital in Bobigny with a febrile illness that lasted 4 days. She had been traveling for 2 months through Senegal and Mauritania. The day of her return to France, high-grade fever (temperature 41°C), chills, headache, diarrhea, and arthralgia developed. Results of a clinical examination were normal. Laboratory investigations showed anemia (hemoglobin 9.8 g/dL) and thrombocytopenia (64,000 platelets/μL). Giemsa-stained blood smears showed spirochetes. Molecular analyses identified this bacterium as *B. crocidurae*. The patient was treated with doxycycline, 100 mg twice a day for 10 days, and the patient quickly recovered.

Relapsing fevers caused by *Borrelia* spp. are rarely reported in travelers from disease-endemic countries. Because most infections are benign, cases are probably neglected. Since cultivation of the causative agents can be difficult, diagnosis relies on microscopic detection of helical bacteria in stained blood smears. Blood samples should be obtained during febrile episodes, but as shown in patient 2, spirochetes may be visualized on blood smears when the patient is no longer febrile. Quantitative buffy-coat analysis that increases detection sensitivity has been reported (*5*). Serologic tests are being developed to diagnose infection with *B. recurrentis* (*6*).

Detection of *Borrelia* DNA by PCR amplification from the blood is highly sensitive and specific. Identification can be achieved by sequencing the 16S rRNA gene (*7*) (Figure). Given the high level of sequence conservation (*7*), mutations can be informative. The identifying nucleotides for *B. crocidurae*, *B. hispanica*, and *B. duttoni* were at positions 65, 181, 381, and 596. Therefore, sequence analysis of the first 600 nucleotides (nt) in the 16S rRNA gene is sufficient to differentiate *B. crocidurae*, *B. hispanica*, and *B. duttonii*.

**Figure:**
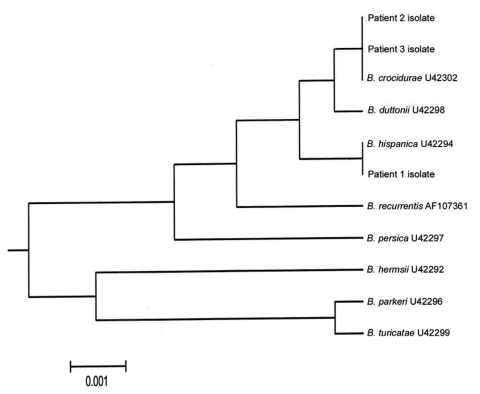
Unweighted pair group with mathematical average rooted tree of complete sequences of the Borrelia 16S rRNA gene. Sequences from databanks are indicated by their accession numbers.

The complete *Borrelia* sequence obtained from the patient 1 showed 99.93% identity with *B. hispanica* (1 nt difference), 99.79% with *B. crocidurae* (3 nt differences), and 99.72% identity with *B. duttonii* (4 nt differences). This patient was infected with *B. hispanica* during his travel through Spain or Morocco, which is consistent with the distribution of this species. Comparison of 1,430 nt from sequences from patients 2 and 3 showed 99.93% identity with the sequence of *B. crocidurae* (1 nt difference). *B. crocidurae* occurs mostly in sub-Saharan countries of West Africa (Senegal, Mali, and Mauritania), where patients 2 and 3 were likely to have been infected.

Physicians should be alert for relapsing fever in travelers, and this diagnosis should be considered in febrile patients from disease-endemic regions. Diagnosis relies upon examination of stained blood smears. Where available, molecular methods are highly efficient to detect and identify bacterial species. Other tickborne infections (e.g., those with *Rickettsia* spp.) should also be considered in patients returning from disease-endemic countries (*8*). The recommended treatment is doxycycline, although it can cause a Jarish-Herxheimer reaction in some patients.
